# The Influence of Muscle Flexibility Training on Chronic Pain in Older Adults: An Exploratory Systematic Review

**DOI:** 10.3390/sports13110393

**Published:** 2025-11-05

**Authors:** Rodrigo Melenas, Raúl Antunes, Rui Matos, Diogo Monteiro, Nuno Amaro, Nuno Couto, Miguel Jacinto

**Affiliations:** 1 ESECS, Polytechnique of Leiria, 2411-901 Leiria, Portugalrui.matos@ipleiria.pt (R.M.); diogo.monteiro@ipleiria.pt (D.M.);; 2 Research Center in Sport, Health, and Human Development (CIDESD), 5000-558 Vila Real, Portugal; 3 Sport Sciences School of Rio Maior, Santarém Polytechnique University (ESDRM-IPSantarém), 2040-413 Rio Maior, Portugal

**Keywords:** flexibility exercise, PNF, older adults, pain

## Abstract

**Background/Objectives:** This exploratory systematic review aims to analyze the influence of isolated muscle flexibility training on the reduction of chronic pain symptoms in older adults aged 65 years or more. Articles were selected from the Web of Science, PubMed, and Scopus databases, using the EndNote software for reference management. The selection process followed the PICOS framework and the PRISMA 2020 guidelines, and the review protocol was registered in the PROSPERO database. **Methods:** The inclusion criteria comprised randomized controlled trials with participants aged 65 or older, evaluating the effect of flexibility training as a standalone intervention on chronic pain, and published in English or Portuguese. Studies were excluded if they involved multimodal training, did not specify participants’ ages, evaluated only acute or postoperative pain, or were not peer-reviewed articles. **Results:** From an initial pool of 1390 articles, only three met all criteria and were included in the final analysis. These studies—conducted in China (n = 2) and the United States (n = 1)—showed moderate methodological quality (PEDro score = 7/10). Two trials applied Proprioceptive Neuromuscular Facilitation (PNF) in participants with knee osteoarthritis, while the third compared a flexibility-based program to combined strength and aerobic training in a healthy elderly population. All studies reported significant reductions in chronic pain symptoms following flexibility training interventions. **Conclusions:** The reviewed evidence suggests that muscle flexibility training, particularly using techniques like PNF, may be a promising therapeutic strategy to mitigate specific chronic pain-related symptoms in older adults, particularly reductions in joint stiffness, movement discomfort, and pain intensity associated with osteoarthritis. However, the limited number of high-quality trials and heterogeneity in protocols and pain assessment tools highlight the need for further research.

## 1. Introduction

Aging is a physiological and multifactorial process, characterized by progressive changes in biological systems, directly and indirectly affecting individuals’ quality of life [1]. In Portugal and most European countries, the population aged 65 years or older is considered elderly [2]. The aging process can be described as accumulation of molecular and cellular damage that the body undergoes over time. This progression leads to a gradual decline in physical and mental capacities, increasing the risk of chronic illnesses, disabilities, and even death [3].

Among the most prevalent and debilitating symptoms in the elderly are chronic pain—defined as an unpleasant sensory and emotional experience that persists for more than three months, which may or may not be associated with identifiable tissue damage [4]. This condition substantially compromises functionality, independence, and psychological well-being, potentially intensifying anxiety, depression, and social isolation. It also leads to increased medication use and higher healthcare costs [4].

The vulnerability of this population is inherently linked to advanced age; however, the presence of pain further aggravates these symptoms and contributes to the onset or worsening of other conditions, increasing the risk of health deterioration [4] and elevating healthcare utilization rates [5]. Furthermore, the use of NSAIDs (Nonsteroidal Anti-Inflammatory Drugs) is observed in 30% of the population aged between 65 and 89 years [6], despite known risks of gastrointestinal bleeding and cardiovascular events in older adults [7]. This practice has financial implications both for individuals and the government (if covered by public healthcare) due to the high costs of managing NSAID-related complications [8]. To put this into perspective, Portugal’s National Health Service spent approximately 3.96 billion euros on medications in 2024—an increase of 11.5% compared to previous years [9], reflecting a global trend of rising pharmaceutical expenditures in aging populations [10].

Given this context, various approaches have been explored as non-pharmacological and more cost-effective strategies for managing chronic pain in older adults. Among them, regular physical activity stands out as one of the most recommended in the scientific literature, being associated with improvements in physical function, mobility, balance, and mental health [11]. Recent evidence further supports that combined exercise and psychological interventions significantly reduce pain intensity and depressive symptoms in this population (differences: −0.34 to 0.54) [12]. Among the different components of physical fitness, muscular flexibility is often underestimated, despite playing a crucial role in maintaining range of motion, preventing joint stiffness, and promoting functional autonomy [13]. Stretching programs, including static and PNF techniques, have been shown to enhance joint mobility and tolerance in older adults [14], while resistance training improves functional capacity (e.g., 6MWT and SUCSD performance) [15]. This type of training involves consciously and controlled stretching of muscle fibers through exercises designed to increase joint range of motion, ultimately enhancing an individual’s ability to move with fewer limitations in daily life [11]. Notably, physical activity may also modulate inflammatory pathways (e.g., IL-10 upregulation) to prevent chronic muscle hyperalgesia [16], and clinical guidelines recommend structured flexibility training ≥2–3 times/week for functional preservation [11,17].

Several factors support the selection of flexibility training for this population, including the potential improvements in range of motion [11] and reduced joint stiffness [18], as well as its low-impact nature, which reduces injury risk compared to high-intensity exercises [19], and minimal-to-no equipment requirements. Improved joint mobility significantly enhances quality of life, allowing elderly individuals to perform daily tasks more comfortably and independently, particularly in those with osteoarthritis [20]. Its low-impact nature makes it a suitable approach for older adults who may require more moderate interventions due to certain health conditions, such as osteoporosis or cardiovascular limitations [21]. Additionally, it is easy to practice without specialized equipment—unlike other exercise types (e.g., running shoes), making it more affordable overall and accessible for low-income populations [22].

The American College of Sports Medicine (ACSM) recommends that older adults engage in regular flexibility exercises to maintain or improve joint range and overall mobility [11]. However, despite evidence linking flexibility to functionality, little is known about the direct impact of specific flexibility training programs on chronic pain as a primary outcome. Existing literature has established the importance of flexibility for functional mobility [23], but few studies have examined flexibility training as a primary intervention for chronic pain. This gap in knowledge limits the development of evidence-based, targeted protocols for this population.

This exploratory systematic review aims to analyze the influence of isolated muscle flexibility exercises on chronic pain levels in older adults. The goal is to contribute to a better understanding of the effectiveness of this intervention and support the development of safe, accessible, and sustainable therapeutic strategies for pain management in this age group.

## 2. Materials and Methods

This systematic review was conducted following the Preferred Reporting Items for Systematic Reviews and Meta-Analyses (PRISMA) guidelines (please see Supplementary Materials), in accordance with the recommendations outlined by Page et al. [24]. The review was also registered in the PROSPERO database. To structure and organize the key components of this study, as well as to define the inclusion and exclusion criteria, the PICOS framework (Population, Intervention, Comparison, Outcomes, Study design) was applied. This approach ensured methodological rigor by guiding the formulation of the research question and the systematic selection of eligible studies.

### 2.1. Study Selection and Research

To select the articles for this systematic review, three electronic databases were used on 21 July 2025: Web of Science (title, abstract, and keywords), PubMed (title and abstract), and Scopus (title, abstract, and keywords). No specific time frame was applied in the search process to include all potentially relevant studies on the topic. A comprehensive search string was developed to capture all articles addressing the following terms: (“Older Adults” OR Elders OR Elderly OR Seniors OR “Geriatric population” OR “Older individuals” OR “Mature adults” OR “Aged” OR “Aging population” OR “Older people”) AND (“Flexibility training” OR “Flexibility exercises” OR “Stretching exercises” OR “Stretching training” OR “Musculoskeletal flexibility” OR “Range of motion exercises” OR “Mobility training” OR “Joint flexibility” OR “Static stretching” OR “Dynamic stretching” OR “ROM exercises” OR “Flexibility intervention”) AND (Pain).

The inclusion criteria for this systematic review were: (1) Population: individuals aged 65 years or older; (2) Intervention: studies using flexibility training as a standalone intervention; (3) Outcome: studies evaluating chronic pain as one of the measured outcomes; (4) Study design: randomized controlled trials (RCTs); (5) Language: studies published in Portuguese and/or English. The exclusion criteria were: (1) Studies involving multimodal training interventions; (2) Studies that did not clearly specify the age of participants; (3) Studies that assessed only acute pain or postoperative pain, rather than chronic pain; (4) Publications that were not peer-reviewed articles; (5) Gray literature.

### 2.2. Data Extraction and Synthesis

All articles retrieved from the electronic databases were exported into a reference management library using EndNote X7 software (Clarivate, Philadelphia, PA, USA). After the removal of duplicates, the titles and abstracts of the remaining studies were independently screened by two reviewers according to the eligibility criteria. Discrepancies between reviewers were resolved through discussion or, when necessary, consultation with a third reviewer. The full texts of potentially eligible studies were then assessed in detail to confirm their inclusion based on the predefined criteria. The entire selection process followed the standard PRISMA flow diagram to ensure transparency and methodological rigor in identifying, screening, and including studies for this systematic review.

This multi-phase selection aimed to minimize bias and enhance reproducibility, strengthening the validity of the final evidence base analyzed.

### 2.3. Assessment of Methodological Quality

To assess the methodological quality of the randomized controlled trials included in this systematic review, the PEDro scale (Physiotherapy Evidence Database) was employed. This is a validated and widely used tool in rehabilitation research that helps evaluate the internal validity and statistical reporting of clinical trials. The scale consists of 11 items, of which 10 are scored (excluding the first item, which pertains to external validity regarding participant eligibility). The criteria assessed include aspects such as proper random allocation, concealed allocation, baseline comparability of groups, blinding of participants, therapists, and outcome assessors, intention-to-treat analysis, adequacy of follow-up (i.e., outcome data obtained for at least 85% of participants), and the presentation of between-group statistical comparisons and measures of variability (e.g., standard deviations or 95% confidence intervals). Each satisfied criterion receives one point, resulting in a total score ranging from 0 to 10. Scores between 6 and 10 are generally interpreted as indicating moderate to high methodological quality. In this review, the PEDro scale was independently applied by two reviewers to each included study. Disagreements in scoring were resolved by consensus, ensuring consistent and reliable quality appraisal. The use of this standardized tool enhances the objectivity and transparency of the risk of bias assessment, thereby contributing to the credibility and robustness of the findings and conclusions drawn from the selected evidence base.

## 3. Results

**Figure 1:**
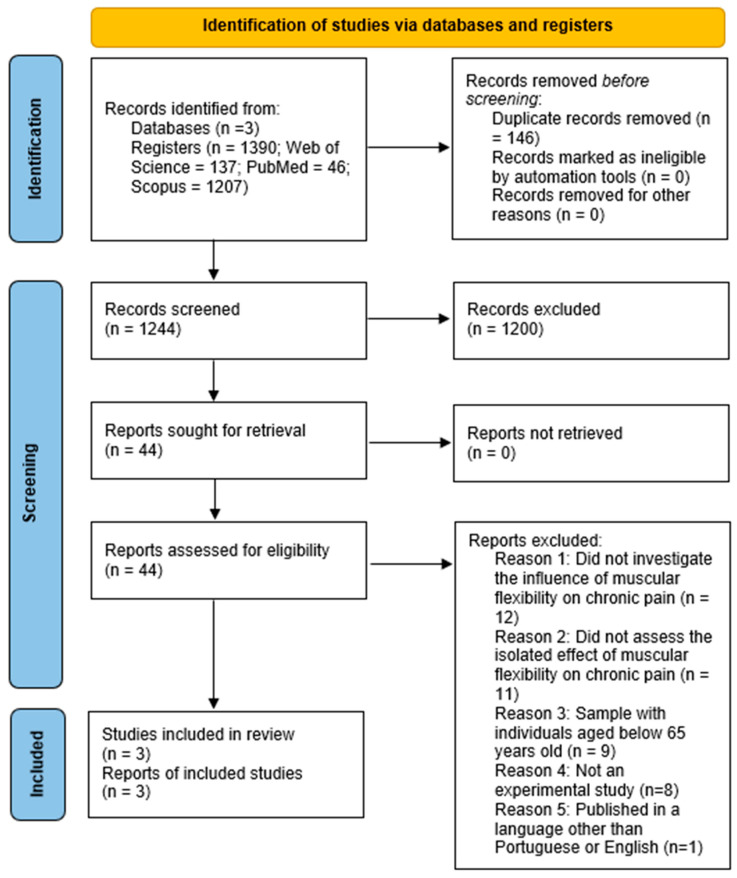
PRISMA 2020 flow diagram for included searches.

Initially, 1390 records were identified in the Web of Science, PubMed, and Scopus databases, of which 146 duplicates were removed, leaving 1244 for screening. After reading the titles and abstracts, 1200 records were excluded, and 44 articles were evaluated in full. Of these, 41 were excluded for failing to meet the inclusion criteria (Most of the excluded studies failed to meet key criteria such as the use of isolated flexibility training, the inclusion of participants aged 65 or older, or the assessment of chronic pain as a primary outcome, using multimodal interventions or lacking methodological rigor (e.g., non-randomized designs or insufficient reporting), resulting in only three eligible studies. The final selection of studies represents the limited but growing body of evidence investigating the isolated effects of flexibility training on chronic pain in the elderly, highlighting the need for further high-quality research in this area. For more information, please see Figure 1.

### 3.1. Methodological Quality

The methodological quality of the studies included in this systematic review was assessed using the PEDro scale. All three studies [25,26,27] received a total score of 7 out of 10, which corresponds to a classification of moderate methodological quality. Common strengths among the studies included appropriate randomization, baseline similarity between groups, intention-to-treat analysis, outcome measures collected from more than 85% of participants, clear reporting of data variability (e.g., mean and standard deviation), and statistical comparisons between groups. On the other hand, the main limitations identified were the lack of allocation concealment and the absence of blinding of participants, therapists, and outcome assessors. These methodological shortcomings may introduce some risk of bias; however, the overall design of the trials demonstrated sound practices in clinical research. Therefore, the findings can still be considered consistent and relevant within the context of the evidence reviewed.

### 3.2. Characteristics of Included Studies

Of the three studies included in this systematic review, two were conducted in China (in 2020 and 2023, respectively), while the third was carried out in the United States in 2000. The sample sizes in the Chinese studies were relatively small, comprising 27 and 29 participants, whereas the American study involved a substantially larger cohort of 103 participants. According to the three studies, the average age of participants was approximately 69.3 years, reflecting a typical older adult population. In both Chinese studies, all participants were clinically diagnosed with knee osteoarthritis, and the severity of the condition was assessed using the Kellgren and Lawrence scale—a widely accepted radiographic classification method for grading osteoarthritic degeneration. In contrast, the U.S.-based study focused on a generally healthy elderly population, with no specific pathological condition reported, though participants were medically screened to exclude those with cardiovascular disease or history of stroke. In terms of gender distribution, all three studies showed a higher proportion of female participants, consistent with demographic trends in aging populations. The intervention protocols in the Chinese studies involved two distinct groups: an intervention group (receiving Proprioceptive Neuromuscular Facilitation—PNF—flexibility training) and a control group (either passive activity or no intervention). These designs allowed for a clear assessment of the isolated effect of flexibility training on chronic pain reduction. Conversely, the U.S. study implemented a comparative approach using two intervention groups: one performing a combined strength and aerobic training program (Fit & Firm), and the other engaging in a flexibility and relaxation program (Stretch & Flex). This structure aimed to evaluate the relative effectiveness of different exercise modalities. Pain measurement tools also differed across studies. The Chinese trials employed the Visual Analog Scale (VAS) and the Western Ontario and McMaster Universities Osteoarthritis Index (WOMAC), both validated instruments widely used in musculoskeletal and osteoarthritis research. The U.S. study utilized scales derived from the Medical Outcomes Study (MOS), which are commonly used to assess general health-related quality of life, including pain dimensions. These variations in methodology, sample characteristics, and assessment tools reflect the heterogeneity among the selected studies, underscoring both the complexity and the potential of investigating the impact of flexibility training on chronic pain in elderly populations. For more information, see Table 1.

### 3.3. Type of Study

The types of studies used as the basis for analysis in this systematic review are referred to as RCTs. These are characterized by the random allocation of individuals into groups. These groups may be referred to as the intervention group (IG), whose participants receive the intervention being studied, and the control group (CG), whose participants do not receive the intervention and serve as a comparison for the intervention group.

Both articles originating from China used one intervention group and one control group to analyze the results of the variable under study. They used the PNF technique, mentioned in the ACSM [11] as a method to work on muscle flexibility. In contrast, the study conducted in the USA featured two intervention groups, where the results of a flexibility exercise program were compared with a distinct program practiced by the other group.

### 3.4. Sample Characteristics

Regarding sample size, the studies conducted by Song et al. [26] and Gao et al. [27] present similar values, with one having 29 individuals (IG = 13; CG = 16) and the other 27 (IG = 13; CG = 14), respectively. The study developed by King et al. [25], on the other hand, included a total of 103 individuals, who were distributed using a computerized version of the Efron procedure after stratification by sex. However, the number of individuals per group after these procedures was not disclosed.

As for average age, King et al. [25] reported an average of 70 ± 4 years. Song et al. [26] presented an average of 68.5 ± 4.3 years (IG) and 67.4 ± 3.4 years (CG). Gao et al. [27] reported an average of 68.54 ± 2.07 (IG) and 67.86 ± 1.41 (CG).

It is worth noting that both studies by Song et al. [26] and Gao et al. [27] involved participants diagnosed with knee osteoarthritis, with the severity of this condition being assessed using the Kellgren and Lawrence scale. Gao et al. [27] reported, in the IG, 6 subjects with grade II (46%), 5 with grade III (38%), and 2 with grade IV (16%). In the CG, 5 subjects had grade II (36%), 7 had grade III (50%), and 2 had grade IV (14%).

For the study by Song et al. [26], the data did not specify the number of participants per severity grade, only indicating they were distributed between levels I and III. The study by King et al. [25] did not specify any common diagnosed condition in the admitted population. However, participants were required to be free from cardiovascular diseases and/or strokes, as confirmed through medical evaluations.

There was also a noticeable predominance of female participants compared to male participants: 65% of the sample in King et al. [25], 62.1% in Song et al. [26], and 59.3% in Gao et al. [27].

### 3.5. Interventions Carried out in Different Studies

As referenced in Table 1 all studies are experimental in nature as they are randomized controlled trials. Both Chinese studies included two groups in which participants were randomly assigned to the IG or CG. The study conducted by King et al. [25], however, used two IGs to compare their outcomes and determine which group achieved better results for the variables under study.

#### 3.5.1. Study by Song et al. (2020) [26]

This study investigated the effectiveness of a flexibility program using the PNF technique to determine its influence on pain perception in patients diagnosed with knee osteoarthritis. The intervention lasted 12 weeks, with sessions held three times per week, each lasting one hour. Four movement patterns were pre-established and performed 5 to 8 times per set, totaling three sets per session.

Participants in the CG were instructed to watch television and read magazines during the IG’s exercise sessions.

#### 3.5.2. Study by Gao et al. (2023) [27]

This study evaluated the effect of a flexibility program using the PNF (Proprioceptive Neuromuscular Facilitation) technique over a period of 8 weeks, with a frequency of three times per week, and a session duration of one hour. The intervention targeted four joints—the hip, knee, ankle, and toes—through four pre-established movement patterns.

#### 3.5.3. Study by King et al. (2000) [25]

As previously mentioned, two IGs were used to determine which would yield more significant improvements, not only in the participants’ physical functionality but also in several general well-being indicators, including pain.

Two approaches were established: “Fit & Firm,” which combined strength and aerobic training, and “Stretch & Flex,” which involved multiple flexibility exercises for each muscle group. Each approach was implemented in two different contexts: group classes and home sessions.

The study lasted 12 months, with four sessions per week for both interventions (2 group classes + 2 home sessions), consisting of one-hour group classes and 40 min home sessions. Participants were contacted throughout the study to ensure adherence to the exercise program, provide encouragement, and resolve any potential issues.

### 3.6. Pain Measurement

In the study conducted by King et al. [25], to evaluate and measure certain health and quality of life parameters (including pain), scales from the Medical Outcomes Study were used. Gao et al. [27] used the VAS. Both studies employed these tools before and after the intervention.

The study by Song et al. [26], on the other hand, used the WOMAC and conducted pain assessments at weeks 0, 6, and 12 (end of the intervention).

### 3.7. Results

After analyzing the data from the various interventions, the following conclusions were drawn regarding participants’ pain perception:

In King et al. [25], the comparison between the two IGs revealed that participants in the flexibility program experienced a greater reduction in pain compared to those in the strength and aerobic training group.

In Song et al. [26], the PNF program was shown to reduce pain perception in patients diagnosed with knee osteoarthritis. The same was confirmed in Gao et al. [27], where the same technique once again demonstrated a reduction in pain for patients with the same condition.

## 4. Discussion

This systematic review with limited evidence aimed primarily to determine whether a standalone flexibility exercise program has any influence on chronic pain in a population aged 65 years and older. Based on the three included randomized controlled trials, the findings suggest that such an approach may indeed contribute to a reduction in chronic pain in older adults, indicating that flexibility-based interventions may be a viable option for managing this symptom. These findings align with broader evidence on exercise and pain management in aging populations [11,28] but also highlight the unique advantages of isolated flexibility training.

The first study, conducted by King et al. [25], compared two exercise approaches applied to a population not characterized by a specific pathology. One approach involved isolated flexibility training through stretching exercises, while the other combined aerobic and strength training. Interestingly, the group that engaged in the flexibility-based program demonstrated greater reductions in chronic pain, suggesting that flexibility exercises may yield effects equal to or even superior to more traditionally recommended forms of training, such as resistance or aerobic exercise. This finding is particularly noteworthy, as it challenges the conventional hierarchy of physical exercise interventions for older populations and highlights the therapeutic potential of targeted flexibility programs. The greater pain reduction in the flexibility group challenges conventional hierarchies favoring multimodal exercise [29]. This echoes recent debates about the underappreciated role of flexibility in pain management in geriatric [23], although the lack of a true control group (e.g., no exercise) limits causal inferences.

The other two studies, conducted in China [26,27], shared similar research frameworks and methodologies, with overlapping research teams and institutions involved. Both studies applied the PNF technique to improve muscular flexibility, targeting elderly populations diagnosed with knee osteoarthritis. These studies confirmed the effectiveness of PNF not only in enhancing joint flexibility but also in reducing pain intensity in this clinical population. The consistent positive outcomes in these studies provide further support for incorporating flexibility training into non-pharmacological pain management protocols for older adults with degenerative joint conditions.

When examining the magnitude of the effects reported in the included trials (Table 1), flexibility-based interventions demonstrated consistent and clinically meaningful reductions in chronic pain among older adults. In Song et al. [26], the PNF program produced a large effect size (η^2^ₚ = 0.789), corresponding to a substantial decrease in WOMAC pain scores from baseline to post-intervention. Similarly, Gao et al. [27] observed a large within-group improvement (Cohen’s d = 1.833) and a moderate between-group difference (*d* = 0.987) favoring the flexibility intervention. Even in the non-pathological sample analyzed by King et al. [25], the flexibility + relaxation program resulted in a significant reduction in chronic pain compared with the strength and aerobic training group (F(4, 98) = 12.29, *p* < 0.001). Collectively, these findings suggest that flexibility training, particularly when delivered through structured PNF techniques, can elicit moderate-to-large reductions in perceived pain intensity. However, the interpretation of these effect sizes must be tempered by the small sample sizes and methodological variability across studies, which may inflate statistical estimates and limit the generalizability of the observed benefits.

Despite these promising findings, the existing literature on isolated flexibility training remains limited. Most research in this domain has focused on multimodal interventions that combine flexibility with other exercise types or adjunct therapies [11]. For instance, Zhu et al. [30] conducted a meta-analysis that examined the efficacy of various interventions, including PNF, for reducing chronic pain in older adults with knee arthritis. Their results further substantiated the potential of PNF, indicating that it had a statistically significant effect in reducing chronic pain in this population (Standardized Mean Difference: 2.54 [95% CI: 1.23; 3.84]) when compared to static stretching and other modalities. Importantly, one of the studies included in their meta-analysis—Song et al. [26]—was also analyzed in the present systematic review with limited evidence, thereby reinforcing the consistency and relevance of these findings. However, the exclusive focus on knee OA raises questions about generalizability to other chronic pain conditions, which also benefit from flexibility training but may require tailored protocols [12].

However, several limitations must be acknowledged. First, the number of high-quality studies focusing exclusively on flexibility exercises is still very limited. Among the studies reviewed, two focused on populations with a specific pathology—knee osteoarthritis—which restricts the generalizability of the findings to the broader older adult population without this condition. Additionally, the studies used different tools for pain assessment, including the Visual Analog Scale, the WOMAC index, and the Medical Outcomes Study instruments. This heterogeneity in outcome measurement complicates direct comparisons and may contribute to variability in effect sizes reported. At the same time, the methodological quality of the included studies was rated as moderate (PEDro score = 7/10), none implemented allocation concealment or participant blinding, which introduces potential bias that should be considered when interpreting the results.

Given these limitations, future research should prioritize increasing the number of randomized controlled trials that examine flexibility training as an independent intervention. Studies involving larger, more heterogeneous, and pathology-free samples are essential to improve the generalizability of findings. Moreover, the use of standardized and validated outcome measures for chronic pain would facilitate more accurate cross-study comparisons, enhance the statistical power of future meta-analyses, and strengthen the reliability of effect estimates and confidence intervals (e.g., 95% CI).

Notwithstanding these limitations, the results from the three studies included in this review have notable practical implications. Flexibility-focused interventions, particularly those based on PNF techniques, offer a non-pharmacological alternative for managing chronic pain in the elderly. This type of intervention is relatively low-cost, does not require expensive equipment, and can be implemented in various settings—from clinical environments to in-home sessions—thus promoting accessibility and adherence among older adults. Moreover, flexibility training presents minimal risk and aligns well with the needs and capacities of aging individuals. These characteristics make it a valuable tool for health professionals and exercise specialists aiming to promote functional independence and quality of life in older populations. Its safety profile is particularly relevant for frail elderly individuals, who face greater risks with pharmacotherapy [31]. However, barriers to adherence (e.g., motivation, mobility limitations) require attention.

In conclusion, isolated flexibility training, especially when delivered through structured techniques such as PNF, demonstrates promise as an effective, practical, and low-risk strategy for managing chronic pain in older adults. While additional research is warranted to confirm and expand upon these findings, the current evidence supports the inclusion of flexibility-based programs in geriatric exercise and rehabilitation protocols.

## 5. Conclusions

The results of this study, although derived from a small number of eligible studies, suggest that flexibility training—especially through proprioceptive neuromuscular facilitation (PNF) techniques—has potential benefits in reducing chronic pain in older adults. However, the scarcity of studies that simultaneously meet the established methodological criteria highlights an important limitation: the current scientific basis is still insufficient to allow for robust generalizations. In this sense, rather than providing definitive conclusions, this study highlights a significant gap in the field, reinforcing the need for further investigation.

Future research should prioritize larger samples, as well as greater standardization of intervention protocols, to enable consistent comparisons between results. In addition, it will be essential to assess the impact of flexibility training in different contexts—clinical and non-clinical—and in association with other therapeutic approaches, such as pharmacological and physiotherapeutic treatments. This will make it possible to build a more solid and clinically relevant evidence base capable of guiding therapeutic decisions and promoting advances in the rehabilitation and quality of life of the elderly population with chronic pain.

## Figures and Tables

**Table 1:** Characteristics of included studies.

Thor (Year)	Country	Sample Size	Mean Age	Clinical Condition Severity	Women (%)	Exercise Program Description	Pain Measurement Tool	Results
King et al. (2000) [25]	USA	N = 103	70 ± 4 years	Healthy population	65%	Intervention group (IG)1: Strength + aerobic training IG2: Stretching + relaxation (Group assignment determined by Efron randomization procedure); 12 months.	Two-item general bodily pain scale from MOS-derived measures	IG2 showed a greater effect compared to IG1 in both sexes in reducing chronic pain. ANCOVA results: F(4, 98) = 12.29, *p* < 0.0007 IG1: Women: Pre = 58.0 (SD = 21.5), Change = −7.0 (SD = 29.6) Men: Pre = 52.8 (SD = 26.7), Change = −2.8 (SD = 21.9) IG2: Women: Pre = 56.6 (SD = 23.5), Change = +7.3 (SD = 26.3) Men: Pre = 63.3 (SD = 20.6), Change = +9.4 (SD = 26.7)
Song et al. (2020) [26]	China	N = 29 IG: 13 Control group (CG): 16	IG: 68.5 ± 4.3 years CG: 67.4 ± 3.4 years	Grade I–III (Knee osteoarthritis)	62.1%	IG: PNF flexibility training CG: Watched TV or read magazines during intervention; 12 weeks.	WOMAC Index	IG showed significant reduction in chronic pain (*p* < 0.001, η^2^ₚ = 0.789) compared to CG. IG pain scores: Week 0: 3.19 (SD = 1.47) Week 6: 1.61 (SD = 1.08; *d* = 1.19) Week 12: 0.55 (SD = 0.48; 2.03) CG pain scores remained stable: Week 0: 3.16 (SD = 1.10) Week 6: 3.24 (SD = 1.47) Week 12: 3.21 (SD = 1.18)
Gao et al. (2023) [27]	China	N = 27 IG: 13 CG: 14	IG: 68.54 ± 2.07 years CG: 67.86 ± 1.41 years	Grade II–IV (Knee osteoarthritis)	59.3%	IG: PNF flexibility training CG: Attended health education lectures; 8 weeks.	VAS	IG showed significant reduction in chronic pain compared to CG. Significant time × group interaction: *p* = 0.031, η^2^ₚ = 0.090 IG: Significant reduction after 9 weeks (*p* < 0.001, *d* = 1.833) IG also had lower pain scores than CG (*p* = 0.018, *d* = 0.987) CG: No significant changes (*p* = 0.375, *d* = 0.266)

## Data Availability

No new data were created or analyzed in this study. Data sharing is not applicable to this article.

## Supplementary Materials

The following supporting information can be downloaded at: https://www.mdpi.com/article/10.3390/sports13110393/s1.
